# SciLinker: a large-scale text mining framework for mapping associations among biological entities

**DOI:** 10.3389/frai.2025.1528562

**Published:** 2025-03-19

**Authors:** Dongyu Liu, Cora Ames, Shameer Khader, Franck Rapaport

**Affiliations:** Target, Disease and Systems Biology, Cambridge, MA, United States

**Keywords:** natural language processing, text mining, relationship extraction, named entities recognition, networks, scientific literature

## Abstract

**Introduction:**

The biomedical literature is the go-to source of information regarding relationships between biological entities, including genes, diseases, cell types, and drugs, but the rapid pace of publication makes an exhaustive manual exploration impossible. In order to efficiently explore an up-to-date repository of millions of abstracts, we constructed an efficient and modular natural language processing pipeline and applied it to the entire PubMed abstract corpora.

**Methods:**

We developed SciLinker using open-source libraries and pre-trained named entity recognition models to identify human genes, diseases, cell types and drugs, normalizing these biological entities to the Unified Medical Language System (UMLS). We implemented a scoring schema to quantify the statistical significance of entity co-occurrences and applied a fine-tuned PubMedBERT model for gene-disease relationship extraction.

**Results:**

We identified and analyzed over 30 million association sentences, including more than 11 million gene-disease co-occurrence sentences, revealing more than 1.25 million unique gene-disease associations. We demonstrate SciLinker’s ability to extract specific gene-disease relationships using osteoporosis as a case study. We show how such an analysis benefits target identification as clinically validated targets are enriched in SciLinker-derived disease-associated genes. Moreover, this co-occurrence data can be used to construct disease-specific networks, providing insights into significant relationships among biological entities from scientific literature.

**Conclusion:**

SciLinker represents a novel text mining approach that extracts and quantifies associations between biomedical entities through co-occurrence analysis and relationship extraction from PubMed abstracts. Its modular design enables expansion to additional entities and text corpora, making it a versatile tool for transforming unstructured biomedical data into actionable insights for drug discovery.

## Introduction

Target identification is a critical early step in the pipeline of drug discovery and usually involves experts from various disciplines. These experts work together to define the disease of interest, explore mechanisms of the underlying pathophysiology, and evaluate targets based on criteria such as efficacy, safety, tissue selectivity, and competitive landscape (Shameer et al., 2017; Morgan et al., 2018). Biomedical literature is a key resource for this endeavor. For example, gene-disease associations reported in scientific publications can guide both therapeutic target identification and credentialing. Genes associated with a given disease in large numbers of research articles are intuitively more likely to be found to be fundamental drivers of disease pathogenesis and, therefore, may be attractive candidates for an efficacious therapeutic intervention (Claussnitzer et al., 2020). On the other hand, genes seldom found to be associated with a given disease in the literature may represent untapped therapeutic opportunities worth further investigation.

Furthermore, insights from literature are not limited to simple direct disease-target associations. Because many diseases involve dysregulation of specific cell types rather than whole tissues or organs, identifying cell type-disease associations from the literature is also an essential resource for target identification. By focusing on the key pathogenic cell types associated with a disease, researchers can gain insight into the underlying molecular and cellular mechanisms, which improves the chances of identifying efficacious targets (Van de Sande et al., 2023). In addition, drug-gene and drug-disease associations from scientific literature provide important information on available therapeutic modalities and drug repurposing opportunities for a particular disease area (Lee et al., 2022). Drugs that have been shown to modulate the activity of genes or pathways associated with the disease of interest represent promising candidates for further investigation (Musa et al., 2018). Integration between these various association types allow the relationships to be inspected in context and can show potential synergies that may otherwise remain invisible when each association type is considered individually.

The sheer volume of the biomedical literature makes it increasingly challenging for researchers to manually extract and synthesize relevant information. By applying natural language processing (NLP)-based computational algorithms for the automated extraction and analysis of knowledge from this vast literature, researchers can efficiently identify trends and connections that might otherwise remain hidden (Simmons et al., 2016). This approach enables a more comprehensive view of disease mechanisms, exposing promising therapeutic targets for further investigation in drug discovery. Such automated methods are essential for leveraging the wealth of information available and overcoming the limitations of traditional literature review strategies.

In this paper, we present SciLinker, a novel NLP-based framework to extract entities and associations, including gene-disease, cell type-disease, drug-disease, and drug-gene associations, from large text compendiums. We have run SciLinker on the entire PubMed abstract corpus and present some of the results, demonstrating its utility in mining valuable knowledge from this extensive collection of scientific text corpora. SciLinker provides a text-derived knowledge analysis stream that can be integrated with multi-omics data and AI algorithms (Lessard et al., 2024), enabling a powerful, multifaceted approach to accelerate target discovery and credentialing by highlighting associations among genes, diseases, cell types, and drugs.

## Background and related work

### Named entity recognition (NER) and normalization (NEN)

Recognizing biomedical entities and concepts in text is often the first step in biomedical natural language processing (BioNLP) applications (Jensen et al., 2006). Named entity recognition (NER) and normalization (NEN) are two crucial steps in this process. NER involves identifying and classifying specific biomedical entities in text, such as genes, proteins, drugs, and diseases. Once identified, the entities are normalized to a standardized terminology or ontology such as Gene Ontology or Medical Subject Headings (MeSH) through the NEN step. This normalization step ensures that different mentions of the same entity are linked to the same nomenclature, enabling seamless data integration and downstream relation extraction among the entities and other BioNLP tasks.

Methods for NER and NEN in biomedical text mining can be divided into four categories: rule-based (Soomro et al., 2017), dictionary-based (Wei et al., 2012; Eftimov et al., 2017), machine learning (ML)-based (including supervised and unsupervised, semi-supervised, and deep learning-based) (Zhu et al., 2017), and hybrid models combining rules, dictionaries, and ML methods (Eltyeb and Salim, 2014). With the recent advances in deep learning and large language models, pre-trained language models have been applied to NER and NEN, including to process PubMed abstracts and PMC full texts. One such text mining tool for annotating biomedical concepts in PubMed abstracts and PMC full-text articles is PubTator (Wei et al., 2019). PubTator applies four different ML and dictionary-based hybrid models to tag genes, diseases, cell line, and species. BERN is another tool that uses high-performance BioBERT NER models which recognize known entities and discover new entities. Various NEN models are integrated into BERN to assign a distinct identifier to each recognized entity (Kim et al., 2019). An updated version BERN2 (Sung et al., 2022) improves BERN by employing a multi-task NER model and neural network based NEN models to achieve faster and more accurate inference.

### Relationship extraction (RE)

Relationship extraction (RE) is the task of identifying relationships between entities extracted from the NER and NEN steps. Traditional approaches for RE in the biomedical domain can be broadly categorized into three types: co-occurrence, rule-based, and ML approaches. Co-occurrence-based models quantify the relationships between entities based on co-occurrence statistics in texts, with the idea that the entities that frequently appear together are more likely to be related (Zhou and Skolnick, 2016; Zhang et al., 2018; Pletscher-Frankild et al., 2015). Rule-based approaches use predefined patterns or rules to identify relationships, often leveraging domain-specific knowledge and linguistic structures (Segura-Bedmar et al., 2011). ML approaches learn to recognize relationship patterns from annotated data. Both rule-based and ML approaches provide a qualitative rather than quantitative approach to RE and require extensive efforts to train and maintain (Song et al., 2015; Mahmood et al., 2016; Hou and Kuo, 2016; Bhasuran and Natarajan, 2018). Deep neural network-based methods such as convolutional neural networks (CNNs), recurrent neural networks (RNNs), and combinations of CNNs and RNNs are commonly used for relation extraction and achieve better performance than traditional ML methods (Emmert-Streib et al., 2020). Recent advances in RE focus on pretrained language models, as studies have shown that these models have achieved state-of-the-art performance for biomedical text mining (Fang et al., 2023). Specifically, BERT-based models such as BioBERT, SciBERT, and PubMedBERT have been successfully used for RE from scientific literature (Bhasuran, 2022).

## Materials and methods

### Text corpora

We used the PubMed abstracts as the text corpus for the SciLinker framework to extract biomedical entities and relationships. This corpus included more than 39 million abstracts (as of September 2024). We downloaded PubMed Baseline 2024 XML files from NCBI’s FTP server^1^, which was released on December 8, 2023. Following this initial download, we retrieved the daily update files^2^ to be processed by the SciLinker Pipeline, batched on a monthly basis.

### SciLinker NLP framework architecture

**Figure 1:**
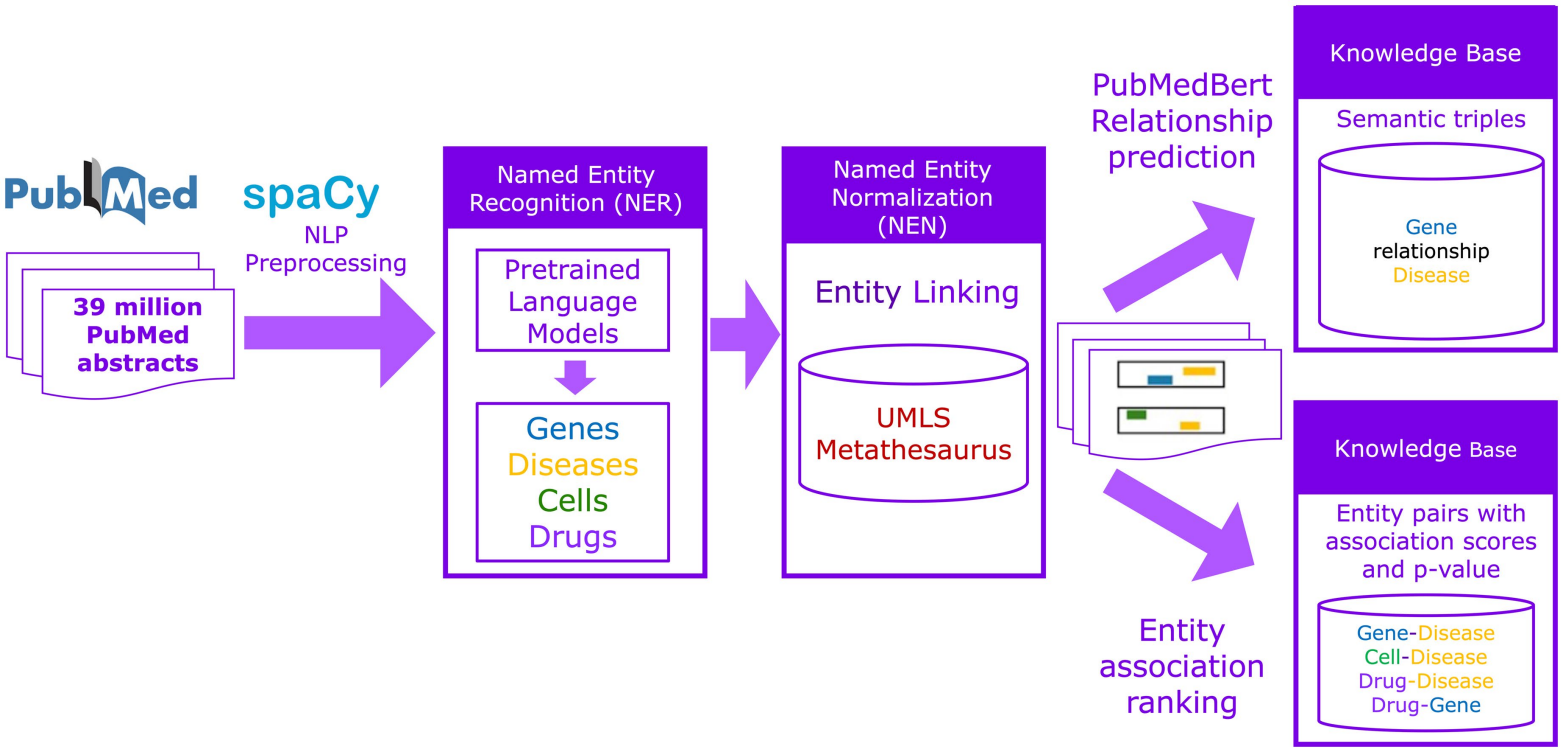
Overview of the SciLinker workflow. SciLinker is a natural language processing (NLP) framework developed using pretrained language models to extract gene-disease, cell type-disease, drug-disease, and drug-gene associations from the PubMed abstract corpus.

We built SciLinker using the open-source NLP libraries spaCy (Honnibal et al., 2020), Stanza (Qi et al., 2020) and scispaCy (Neumann et al., 2019). Stanza is a powerful and efficient Python NLP library providing a comprehensive suite of tools for common NLP tasks. ScispaCy is a Python framework for processing biomedical, scientific, and clinical text. It is built on spaCy, a robust Python library for general domain natural language processing (Figure 1). The preprocessing steps of the pipeline are the following:

- Tokenization – breaking text into word and punctuation tokens.
- Part-of-speech (POS) tagging – assigning POS tags like noun, verb, adjective.
- Dependency parsing – identifying syntactic relationships between words.

### Named entity recognition (NER)

Stanza’s NER model is based on a BiLSTM-CNN-Char framework. This architecture combines bidirectional Long Short-Term Memory (BiLSTM) networks with Convolutional Neural Networks (CNNs) and character-level embeddings to effectively capture both word-level and character-level features for accurate entity recognition (Zhang et al., 2021). SciLinker uses two pretrained biomedical NER models from Stanza for entity recognition tasks. The model trained on the BC5CDR dataset is used to identify diseases and drugs (F1 score of 88.08). The model trained on the BioNLP13CG dataset, which can identify over 14 biomedical entities with F1 score of 84.34 (Zhang et al., 2021), is used to identify genes or gene products (e.g., proteins) and cell types.

### Named entity normalization (NEN)

After the NER step, we normalized entity mentions recognized by the NER models, since the same concept could be represented with different names in different texts. We employed the EntityLinker module from scispaCy to perform NEN, using the Unified Medical Language System (UMLS) knowledge base (Bodenreider, 2004) as the common dictionary. UMLS is a comprehensive thesaurus and ontology for biomedical and clinical domain with a collection of over 200 vocabularies containing 3 million concepts. The NEN step of SciLinker uses character-level 3-grams and Approximate Nearest Neighbors (ANN) to improve the efficiency and accuracy of linking entity mentions to concepts in the UMLS Metathesaurus. Leveraging character-level 3-grams and ANN allow for a fast and scalable search, reducing the computational complexity of the entity linking process so that SciLinker can efficiently compare the character-level representations of entity mentions and candidate concepts.

### Relation extraction

We built a relationship extraction model by fine-tuning the PubMedBERT base uncased version with the balanced training dataset from the paper (Milošević and Thielemann, 2023) for gene-disease associations only. Relation extraction classifies the relationships between named entities in the given text (in this case, gene-disease associations). We followed the preprocessing method used by PubMedBERT (Gu et al., 2020), where entity names are replaced by dummy tokens (e.g., gene and disease names are replaced by $gene and $disease respectively). The model was fined-tuned with the training dataset for 6 epochs (learning rate = 0.00002). The training data contains the following seven gene-disease relationship types (Milošević and Thielemann, 2023):

- No Explicit Relationship – There is no explicit relationship between gene and disease.
- Plays a role – There is a connection between the gene and disease, but the exact relationship is unclear.
- Target → General – The gene can be considered a target for the disease.
- Target → Cause – The gene causes the disease when activated/mutated/inhibited.
- Target → Modulator → Decrease Disease – The gene decreases or alleviates the disease.
- Target → Modulator → Increase Disease – The gene increases or worsens the disease.
- Biomarker – The presence/absence of the gene/protein is an indicator for the diagnosis of disease.

### Co-occurrence scoring

SciLinker provides a co-occurrence-based association score: if an entity pair co-occurs in the same sentence, we consider them to be associated. SciLinker scores the strength of the association using a scoring scheme inspired by the co-occurrence-based text mining scores in the STRING database (Mørk et al., 2014). For an entity pair $\left(x,y\right)$ , x being an entity of type X (e.g., gene) and y an entity of type Y (e.g., disease), we formulate the co-occurrence score $S\left(x,y\right)$ as:


$$S\left(x,y\right)=C{\left(x,y\right)}^{a}{\left(\frac{C\left(x,y\right)C\left(*,*\right)}{C\left(x,*\right)C\left(*,y\right)}\right)}^{1-a}$$

where $C\left(x,y\right)$ denotes the number of times x and y co-occur in the same sentence, $C\left(*,*\right)$ the total number of sentences that contain any entity pair of types X and Y in the text corpus, $C\left(x,*\right)$ the number of sentences that contain both x and an entity of type Y, $C\left(*,y\right)$ the number of sentences that contains both y and an entity of type X, and a the weighting factor. The scoring function therefore corrects the number of co-occurrences by the background distribution of each entity x and y , with a being a trade-off parameter adjusting for the strength of this correction. We propose a=0.6 based on the Mørk et al. (2014) paper.

### Co-occurrence statistical significance

We used a hypergeometric test to determine the probability of observing a certain number of sentences that contain both entities x and y by chance. This assessment considers the total number of sentences in the corpus and the individual frequency of each entity. The hypergeometric test calculates the probability of observing k or more successes (sentences containing both x and y ) in a sample of size *N*, drawn without replacement from a population with K successes and n items of interest. In the context, this is formulated as:


$$P\left(k\right)=\frac{\left(\begin{array}{c} K\\ k \end{array}\right)\left(\begin{array}{c} N-K\\ n-k \end{array}\right)}{\left(\begin{array}{c} N\\ n \end{array}\right)}$$

Where N is the total number of sentences in the corpus that contain both an entity of type X and an entity of type Y, K is the number of sentences that contain both x and an entity of type Y, n is the number of sentences that contain both y and an entity of type X, k is the observed number of sentences that contain both x and y , and $P\left(k\right)$ is the probability that this number is greater or equal to k .

By calculating the hypergeometric probability, we can determine whether the observed association ( k ) between the pair $\left(x,y\right)$ is statistically significant, given the expected distribution of successes based on the total number of sentences, the number of sentences containing x , and the number of sentences containing y . To correct for the multiple comparisons, we adjusted the *p*-value with the Benjamini-Hochberg procedure (Benjamini and Hochberg, 1995).

### Using SciLinker to process PubMed abstracts

We used 10 c6i.8xlarge EC2 instances (32 CPU, 64G RAM) on AWS to run SciLinker on both the PubMed abstract baseline and the daily update XML files. The total running time to process 1,485 XML files with about 39 million abstracts is 7 days. Additionally, we update the SciLinker results with the new daily update files at the end of each month. These monthly updates require less than 2 h of running time on a single instance for about 30 K abstracts on average.

### Fisher’s exact test

We obtained a list of 112 clinically validated psoriasis targets from Citeline^3^, including targets with an approved drug and targets with at least one drug under active clinical development. We categorized these gene targets into four groups based on their clinical development status: phase 1, phase 2, phase 3 and approved. We identified 2,531 psoriasis associated genes from SciLinker. To assess the enrichment of genes under clinical development in these psoriasis-associated genes, we employed Fisher’s exact test. We conducted the Fisher’s exact test using a 2×2 contingency table, comparing the frequency of clinically developed genes in the psoriasis-associated gene set to their frequency in the full set of 19,969 human protein-coding genes (Nurk et al., 2022). Our null hypothesis assumed that there is no association between a gene’s presence in the psoriasis-associated set and its clinical development status. The alternative hypothesis was that genes in the psoriasis-associated set are more likely to be under clinical development for psoriasis treatment. We considered results statistically significant at adjusted *p* < 0.05. We also performed the same test with the clinically validated targets for six other diseases including asthma, atopic dermatitis, COPD, Parkinson’s disease, rheumatoid arthritis (RA), and ulcerative colitis (UC).

### Gene set enrichment analysis

We performed gene set enrichment analysis using the GSEApy package (Fang et al., 2023) in the Python environment. We ran the GSEAPreranked module with the results of disease associated genes for the seven diseases listed in the above section. We used the four clinically validated disease target gene groups described above as gene sets. We compared the GSEA results ranked by SciLinker score vs. ranked by the co-occurrence counts. Normalized enrichment score (NES), *p*-value, and false discovery rate (FDR) for all variables and signatures were obtained in the python environment.

## Results

### Entities and associations output from SciLinker

In this paper, we present a new natural language processing framework named SciLinker to extract four biomedical entities (genes, cell types, drugs, and diseases), as well as gene-disease, cell type-disease, drug-disease, and drug-gene associations from the literature.

The application of the SciLinker framework to the entire PubMed abstract corpus (as of 09/04/2024) resulted in over 11-million gene-disease co-occurrence sentences. These co-occurrences represented associations between more than 29 thousand genes and 16 thousand diseases, giving rise to more than 1.25 million unique gene-disease associations (Table 1), with about 500 thousand found significant (adjusted *p* < 0.05). We also extracted co-occurrence sentences for more than one thousand cell types and 12 thousand diseases. The number of unique cell type and disease association is about 179 thousand, with about half of them found significant (adjusted *p* < 0.05). In addition, we extracted about 32 thousand drugs with about 1 million drug-gene and 839 thousand drug-disease associations.

**Table 1:** Number of entitles and associations extracted by SciLinker.

Entity	Association
Number of diseases	16,413
Number of genes	29,010
Number of cell types	1,586
Number of drugs	32,171
Number of unique gene disease associations	1,250,646
Number of unique cell disease associations	179,042
Number of unique drug disease associations	839,221
Number of unique drug gene associations	1,030,267
Number of gene disease association evidence sentences	11,745,842
Number of cell disease association evidence sentences	3,082,286
Number of drug disease association evidence sentences	8,260,048
Number of drug gene association evidence sentences	8,902,059

### Relationship extraction with PubMedBERT

We performed relationship extraction on gene-disease associations. We obtained a balanced training dataset for gene-disease relationships from Milošević and Thielemann (2023). In that paper, the authors defined seven gene-disease relationship types (Materials and Methods). We fine-tuned PubMedBERT with the training dataset over 6 epochs and obtained an overall weighted average F1-score of 0.88 (Table 2). ‘No Explicit Relationship category’ had a lower F1-score due to fewer training examples. Overall, we achieved F1-scores comparable to that of the Milošević and Thielemann (2023) paper.

**Table 2:** Results of the fine-tuned PubMedBERT model for relationship classification (after 6 epochs).

	Precision	Recall	F1-score
Overall (weighted average)	0.88	0.88	0.88
No explicit relationship	0.57	0.25	0.35
Target → Modulator → Increase disease	0.95	0.91	0.93
Target → Causative	0.91	0.97	0.94
Target → Modulator → Decrease disease	0.83	0.94	0.88
Plays a role	0.90	0.83	0.86
Target → General	0.85	0.95	0.89
Biomarker	0.97	0.95	0.96

We applied the fine-tuned PubMedBERT model to predict gene-disease relationships from 18,136 sentences where both a gene and the disease “osteoporosis” is mentioned. Table 3 shows the percentage of sentences predicted into each relationship type for 47 osteoporosis-associated genes with over 50 co-occurrences. The “Plays a role” has the highest overall percentage across most genes, suggesting that many sentences describe the genes playing a role in osteoporosis without specifying a more detailed relationship. The “Target → General” category also has a notable presence for many genes, indicating that these genes are mentioned as potential targets for treatment or modulation in the context of osteoporosis. The category “No Explicit Relationship” generally has lower percentages across most genes, suggesting in some sentences gene and disease are mentioned together but are not associated with each other. In the more informative category of relationships such as “Target → Causative,” some genes like *WNT1* (53.8%), *PLS3* (49.4%), and *LRP5* (28.9%) have overall high percentages, suggesting that genes are frequently mentioned as potential causative factors for osteoporosis. In the “Biomarker” category, genes such as *POLK* (24.4%), *SLPI* (13.7), *COL1A2* (13.7%), and *SPP1* (12.7%) have a higher percentage, indicating that they are often discussed as potential biomarkers for osteoporosis. Genes playing various roles in bone metabolism, formation, and metabolism such as *NFATC1, MTOR, PPARA, SIRT1* are in the “Target → Modulator → Decrease Disease” category, although there are some conflicting predictions in the “Target → Modulator → Increase Disease” category. In summary, we showed that the fine-tuned PubMedBERT model can be applied to extract gene-disease relationships.

**Table 3:** Percent of each gene-disease relationship category assigned by the fine-tuned PubMedBert model for the 50 osteoporosis-associated genes with 50 or more supporting sentences.

Gene	No explicit relationship	Plays a role	Target → general	Biomarker	Target → causative	Target → Modulator→ Decrease disease	Target → Modulator → Increase disease	Sentence count
TNFSF11	5.5	34.6	30.7	0.5	14.7	12.7	1.4	858
PTH	3.7	31	50.4	2.5	2.2	9.1	1.1	854
TNFRSF11B	5.1	60.2	12.4	2	8.8	9.5	2	693
VDR	0.6	77.1	12.5	1.4	0.9	6.4	1.2	345
SOST	4.3	31.8	50	3.4	0.9	9.3	0.3	324
INS	3.6	63.2	14.6	0	12.6	3.6	2.4	253
IL6	6.1	64.6	10.2	5.3	4.9	5.7	3.3	246
TNFRSF11A	4.9	40.3	37.4	0	8.7	6.8	1.9	206
LRP5	1	60.8	6.9	0	28.9	1.5	1	204
IGF1	4	69.9	16.8	1.2	4	2.9	1.2	173
RUNX2	9.5	37.3	20.1	0	5.3	19.5	8.3	169
EREG	6	35.7	37.5	1.2	3.6	14.9	1.2	168
COL1A2	8.7	58.4	9.3	13.7	6.2	3.7	0	161
CYP19A1	3.8	19.7	40.1	1.3	8.3	20.4	6.4	157
AKT1	10.8	25.7	33.8	0	3.4	25.7	0.7	148
SLPI	18.5	43.8	10.3	13.7	1.4	11.6	0.7	146
TNF	3.3	62.6	13.8	2.4	8.9	8.9	0	123
TGFB1	5.1	63.2	14.5	4.3	4.3	7.7	0.9	117
CTSK	3.6	16.4	69.1	2.7	0.9	7.3	0	110
GH1	0.9	40.9	29.1	0	15.5	10.9	2.7	110
LEP	6.4	66.1	6.4	4.6	1.8	13.8	0.9	109
ESR1	1.9	71.8	16.5	1.9	2.9	4.9	0	103
TNFRSF1A	14.6	44.8	10.4	5.2	6.3	12.5	6.3	96
SIRT1	6.3	20	42.1	1.1	0	26.3	4.2	95
PIK3CA	17.4	23.9	32.6	0	2.2	22.8	1.1	92
EPB42	0	47.2	11.2	6.7	5.6	29.2	0	89
GABPA	5.6	18	29.2	0	3.4	41.6	2.2	89
PLS3	0	48.3	1.1	0	49.4	1.1	0	89
POLK	11.5	29.5	26.9	24.4	1.3	6.4	0	78
COL1A1	1.3	92.2	0	1.3	5.2	0	0	77
BGLAP	12.5	55.6	2.8	19.4	1.4	8.3	0	72
PTHLH	1.4	22.5	57.7	2.8	7	7	1.4	71
SPP1	7	56.3	8.5	12.7	4.2	11.3	0	71
CRP	5.8	66.7	2.9	20.3	2.9	1.4	0	69
AR	1.5	30.9	57.4	1.5	1.5	5.9	1.5	68
DKK1	13.2	45.6	27.9	5.9	1.5	2.9	2.9	68
PPARA	6	26.9	34.3	0	1.5	26.9	4.5	67
NFATC1	7.7	10.8	41.5	0	7.7	32.3	0	65
WNT1	0	40	6.2	0	53.8	0	0	65
ALB	9.4	57.8	10.9	7.8	1.6	12.5	0	64
MIR21	3.2	52.4	15.9	7.9	1.6	19	0	63
SHBG	0	76.2	4.8	4.8	0	4.8	9.5	63
VEGFA	3.2	58.1	16.1	0	4.8	16.1	1.6	62
ESR2	1.7	35	50	3.3	0	10	0	60
MTOR	3.4	13.8	37.9	0	15.5	29.3	0	58
ADIPOQ	14.3	67.9	5.4	5.4	1.8	1.8	3.6	56
BMP2	9.1	47.3	23.6	0	0	18.2	1.8	55
KL	1.9	44.4	7.4	1.9	29.6	13	1.9	54
SP1	1.9	90.4	0	1.9	0	3.8	1.9	52

Percentage is calculated from the total sentences count of each gene.

### Application of SciLinker in target identification

To illustrate how we can apply the SciLinker results for target identification, we will elaborate on two examples. In the first example we looked at *GBA* associated diseases. The *GBA* gene encodes the lysosomal enzyme glucocerebrosidase (GCase), which is responsible for maintaining glycosphingolipid homeostasis. Mutations in the GBA gene can cause Gaucher disease. Approximately 5–15% of Parkinson’s disease (PD) patients have mutations in the *GBA* gene, making it numerically the most important genetic risk factor for PD (Smith and Schapira, 2022). As expected, SciLinker identified Gaucher disease and PD as the top diseases associated with *GBA* (Table 4). Interestingly, the number of co-occurrences between *GBA* and PD was more than with Gaucher disease (78 sentences vs. 76 sentences). However, the association score for Gaucher disease was much higher (284.25 vs. 94.25), due to the background rate correction that is incorporated into the SciLinker association score (see Materials and Methods). Indeed, PD is more often studied in the scientific literature than Gaucher disease (36,946 vs. 1,701 sentences extracted from PubMed abstracts). The same rationale applies for Hemochromatosis and other diseases.

**Table 4:** Output of top diseases associated with GBA from SciLinker.

Disease	Disease CUI	Count	Association score	*P*-value	*P*-adj
Gaucher disease	C0017205	76	284.25	<0.001	<0.001
Parkinson disease	C0030567	78	92.25	5.08E-09	1.29E-06
Gaucher disease, type 3 (disorder)	C0268251	2	40.94	1.12E-07	2.85E-05
Hemochromatosis	C0018995	15	39.89	<0.001	<0.001
Gaucher disease, type 1	C1961835	3	37.47	<0.001	<0.001
Lewy body disease	C0018995	11	36.17	<0.001	<0.001
Synucleinopathies	C5191670	6	34.78	<0.001	<0.001
Presenile dementia	C5191670	12	17.87	<0.001	<0.001
Multiple system atrophy	C0393571	2	11.03	9.67E-05	0.025
Spastic paraplegia	C0037772	2	10.59	0.00012	0.030
Neurodegenerative disorders	C0524851	5	6.83	0.00011	0.028

In the second example, we demonstrate that SciLinker scores can be used to identify potential novel disease targets that would not be obvious using a simple co-occurrence sentence count. Osteoporosis is a condition characterized by weakened bones and an increased risk of fractures, often due to decreased bone density and quality. As the population ages, the prevalence of osteoporosis rises, highlighting the urgent need for new treatments that can effectively prevent bone loss, enhance bone strength, and reduce fracture risk (Li et al., 2021). We applied SciLinker to extract the osteoporosis associated genes from PubMed abstracts. As expected, SciLinker was able to retrieve top osteoporosis associated genes (Supplementary Table 1). Many of the genes are involved in influencing bone metabolism, formation, and resorption. Key genes like *TNFSF11 (RANKL), TNFRSF11B (OPG), PLS3, SOST, LRP5*, and *RUNX2* play crucial roles in bone health, with mutations or dysregulation leading to increased bone fragility and osteoporosis. To identify potential new therapeutic targets, we focused on genes with few co-occurrences with osteoporosis but relatively high association scores. FTCDNL1 ranked 58th on the list by SciLinker score and co-occurred with osteoporosis in only eight sentences. However, this association is highly significant (*p* = 3.82E-07). FTCDNL1 encodes a protein involved in the regulation of bone homeostasis, and its activity influences bone density and strength. Polymorphisms of the FTCHNL1 gene are associated with a reduced risk of having osteoporosis in Asian population, suggesting a potential therapeutic target for osteoporosis (Lu et al., 2015). *FTCDNL1* would rank 519th by sentence counts. This example demonstrates that ranking associations by SciLinker score, and *p*-value can identify statistically significant associations with limited publications, facilitating the discovery of potential untapped therapeutic targets.

### Enrichment of clinically validated drug targets in disease-associated genes from SciLinker results

The goal of target identification is to find potential drug targets that will be successful in clinical trials. To statistically evaluate whether disease associated genes identified through SciLinker were enriched for clinically validated drug targets, we performed Fisher’s exact tests for the disease associated genes from SciLinker for seven diseases (Materials and Methods), since there are only a small number of clinically validated targets available for osteoporosis, we will focus psoriasis as an example in this section. We compared psoriasis associated genes from SciLinker against all human protein coding genes in terms of their clinical development status for psoriasis drugs. We assessed whether the proportion of clinically trialed drug targets in the text-mined list was significantly higher than expected by chance through a Fisher’s exact test (Materials and Methods). Seventy-one of the 2,513 psoriasis associated genes are clinical targets (Odds ratio = 12.22, *p*-value = 3.62E-36). This significant overrepresentation highlights that SciLinker prioritized genes with strong evidence of therapeutic relevance for psoriasis. The Fisher’s exact tests results for the six other diseases also show significant overrepresentation of the clinically validated targets (Materials and Methods).

**Figure 2:**
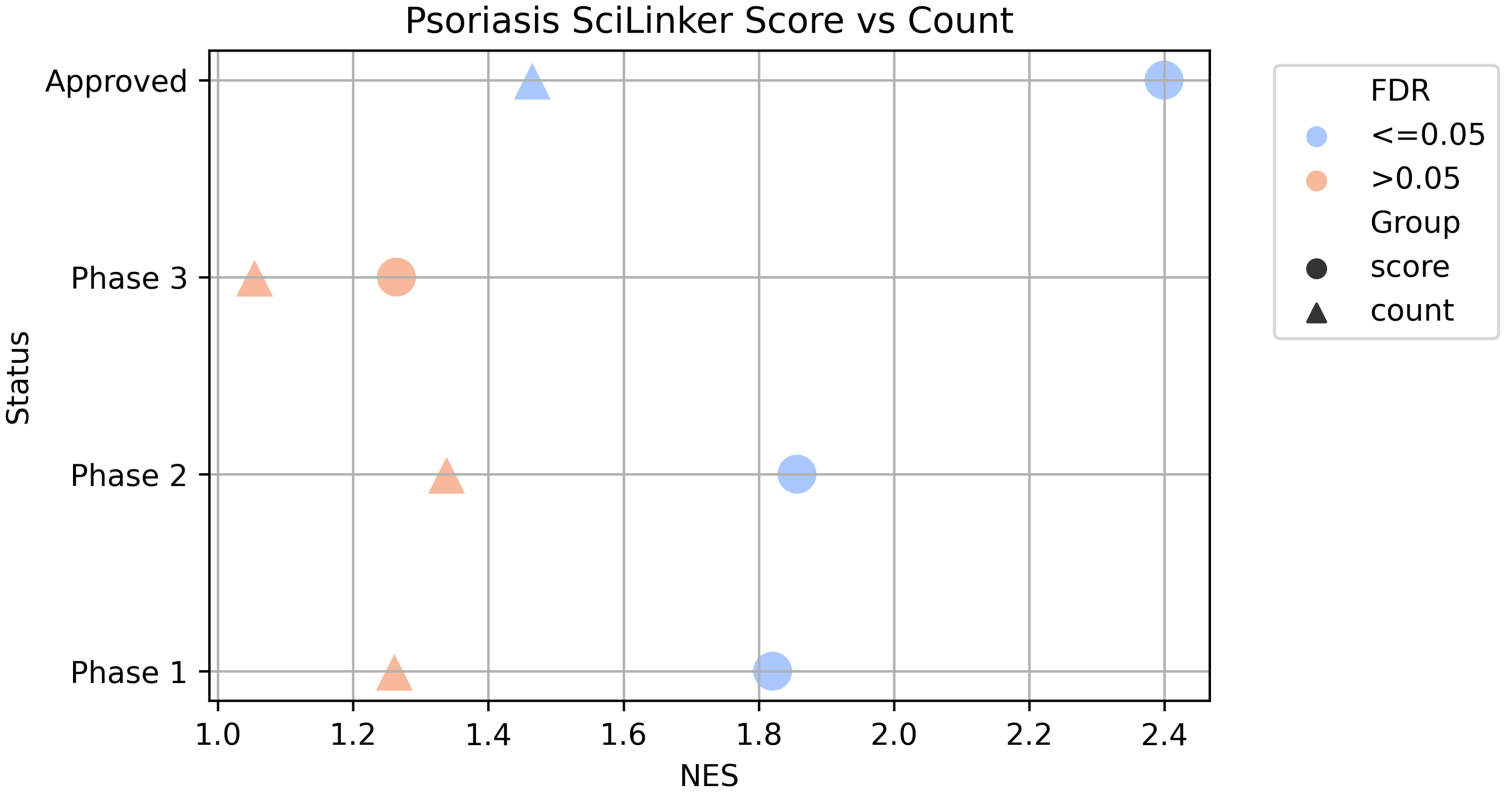
GSEAPreranked results of psoriasis associated genes ranked by SciLinker score vs. ranked by co-occurrence counts. The gene sets are the four clinically validated asthma target groups (phase 1, phase 2, phase 3, and approved). The markers are colored by the FDR.

To further determine whether the disease-associated genes correlate with specific groups of clinically validated targets, we divided the clinical validated targets of each of the seven diseases into four groups based on their clinical status: targets with FDA-approved drugs, targets with drugs in phase 1, phase 2, or phase 3 of their clinical development. We then applied Gene Set Enrichment Analysis (GSEA) using the GSEAPreranked method (Subramanian et al., 2005) to test if any clinical target groups showed statistically significant enrichment in each of the disease-associated gene list ranked by SciLinker scores. Figure 2 shows the results for psoriasis. All four clinical target groups are enriched in the psoriasis-associated genes, but to varying degrees. The approved, phase 2, and phase 1 groups displayed strong enrichment with normalized enrichment scores (NES) of 2.40, 1.86, and 1.82, respectively. The phase 3 group showed weaker, non-significant enrichment with NES around 1.26 (adjusted *p*-value = 0.18). When doing the same analysis with uncorrected sentence counts, the results show all four groups are weakly enriched (NES 1.46, 1.05, 1.34, and 1.33) while only the approved group is significant (adjust *p*-value = 0.03). This demonstrates that the SciLinker scoring approach captures a stronger signal for prioritizing drug targets compared to simple co-occurrence counts.

In summary, we demonstrated that clinically validated targets from all development phases are enriched in the text mining-derived psoriasis genes, with the strongest enrichment seen for approved targets and phase 1–2 trials. We performed the same test for six other diseases (Materials and Methods), with consistent results, showing strong enrichment of clinical targets within SciLinker score ranked genes (Supplementary Figure 1). The correlated enrichment patterns further support the validity of the literature-based disease gene associations.

### Construction of robust network graphs

Co-occurrence data extracted from SciLinker can also be used to construct robust network graphs that capture the relationships among biological entities such as genes, diseases, cell types, and drugs.

We employ the co-occurrence association scores from SciLinker to filter and weight the edges connecting nodes in the network graphs. We can remove edges representing associations that do not meet a specified *p*-value threshold, and the remaining edges have their weights derived from the SciLinker association scores. This approach filters out potential noise while prioritizing robust associations even when raw co-occurrence counts may be lower, thereby highlighting the most statistically significant relationships. Similarly, we can construct weighted and filtered networks for cell type-specific gene expression, drug-target interactions, and other entity relationships using the SciLinker scores and *p*-values.

**Figure 3:**
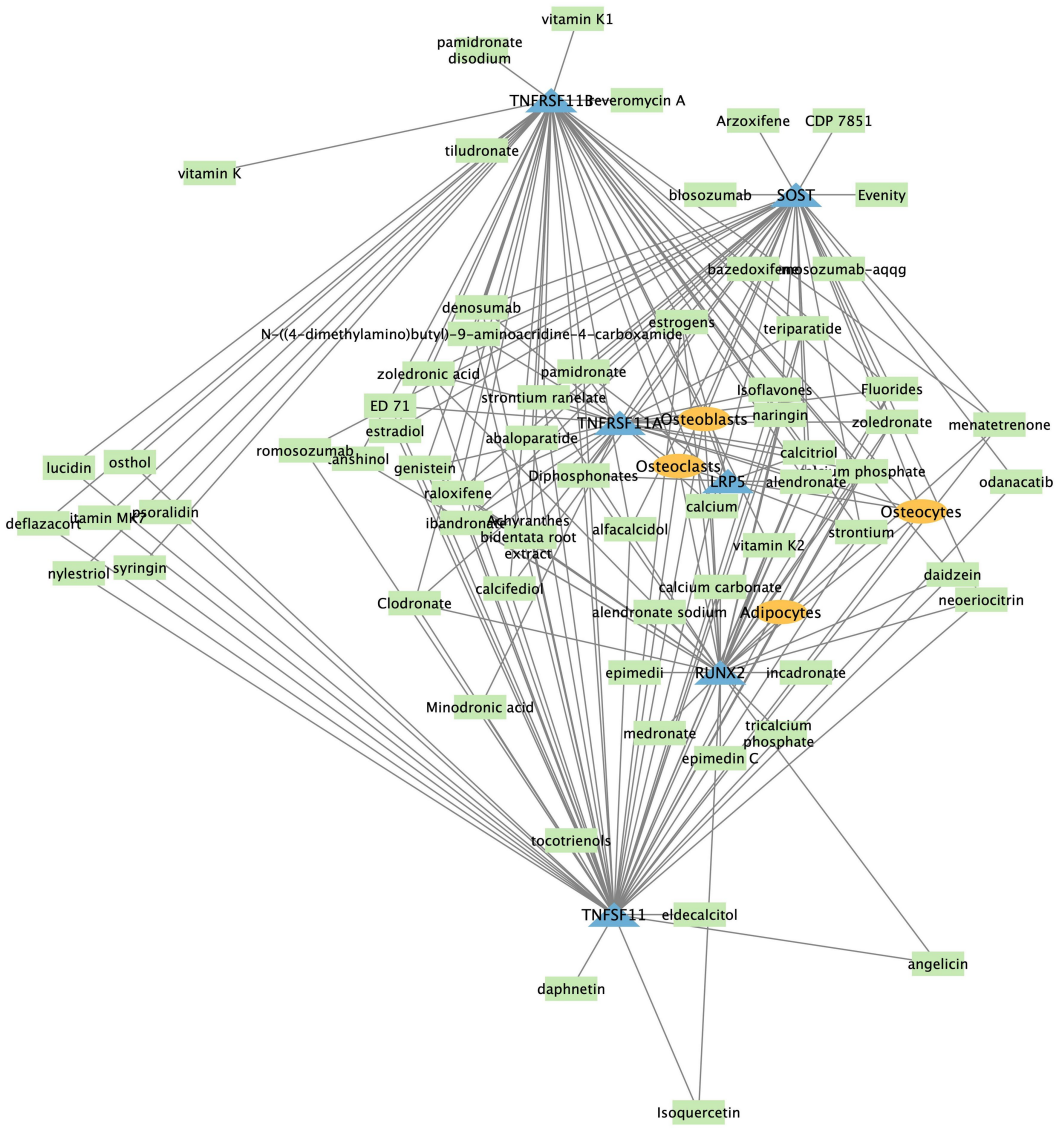
Section of osteoporosis network graph showing the interaction among gene/protein, cell type and drugs. Entities in blue triangle are genes, in green rectangle are drugs, and orange oval are cell types.

We show an example network center around *TNFSF11* for osteoporosis in Figure 3, where significant associations were displayed with a cut-off on adjusted p-value less or equal than 0.05. The network illustrates interactions among genes, drugs, and cell types associated with osteoporosis. The *TNFSF11* (*RANKL*) network is central to osteoporosis, involving key interactions with genes, cell types, and drugs. *RANKL* binds to *RANK* (encoded by *TNFRSF11A*) on osteoclasts, promoting their differentiation and bone resorption, while OPG (encoded by *TNFRSF11B*) acts as a decoy receptor to inhibit this process. Drugs like Denosumab target *RANKL* to reduce osteoclast activity and bone loss. Other genes such as *SOST, LRP5*, and *RUNX2* influence bone formation and remodeling, interacting with the *RANKL* pathway. The balance between osteoclasts, osteoblasts, and osteocytes, along with the modulation by drugs, is crucial for maintaining bone health and treating osteoporosis.

In summary, the high precision co-occurrence disease-specific network, constructed using SciLinker’s robust scoring system and significance testing, empowers data-driven exploration and discovery of only most statistically significant relationships within the vast scientific literature.

## Discussion

SciLinker represents a novel text mining approach for extracting biomedical relationships from scientific literature, specifically focusing on gene-disease, cell type-disease, drug-disease, and drug-gene associations. Using a fine-tuned PubMedBERT model, we demonstrated successful gene-disease relationship extraction across seven relationship types. While SciLinker can identify drugs and cell type entities from PubMed abstracts, we adopted a modular approach and focused primarily on co-occurrence-based relationship extraction due to the significant cost of developing machine learning training datasets for relationship extraction tasks (Milošević and Thielemann, 2023). Our approach effectively identifies significant co-occurring entity pairs in PubMed abstracts and quantifies association strength using both a numerical score and hypergeometric test-based *p*-value.

Several other methodologies exist for extracting biological semantic triples and entity pairs from PubMed abstracts. Milošević and Thielemann (2023) developed a knowledge graph framework using a rule-based system for named entity recognition and normalization (Gerner et al., 2010), achieving an F1-score of 0.92 for gene-disease relationships. Bhasuran and Natarajan (2018) employed joint ensemble learning for gene-disease relationship classification, reaching F1-scores of 0.84–0.87. Our PubMedBERT-based model achieved a comparable average F1-score of 0.88. Importantly, SciLinker extends beyond previous approaches by utilizing state-of-the-art pretrained language models to identify and normalize multiple entity types (genes, diseases, drugs, and cell types) and their co-occurrence associations, enabling the construction of high-precision disease-specific networks.

While other methods like Grissa et al. (2022) and Kim et al. (2017) focused exclusively on gene-disease associations, SciLinker offers broader capabilities. Grissa et al. (2022) used Tagger software (Pafilis et al., 2013) for entity recognition, while Kim et al. (2017) developed DigSee for extracting gene-disease sentences and genetic events. SciLinker distinguishes itself through its comprehensive entity coverage and robust evaluation metrics, combining both association scores and hypergeometric *p*-values to assess relationship significance.

SciLinker’s effectiveness stems from its ability to analyze co-occurrence statistics across large literature corpora, enabling reliable extraction of biomedical associations even with limited context. The framework effectively identifies links for both common and rare diseases, as demonstrated by its successful identification of GBA gene associations with both Gaucher disease (rare) and Parkinson’s disease (common). Importantly, SciLinker can also identify potential novel therapeutic targets, such as FTCDNL1 for osteoporosis. Furthermore, SciLinker’s ranked target lists for diseases show a significant enrichment of clinically validated targets.

SciLinker’s current capabilities can be further expanded in several ways. First, while the fine-tuned PubMedBERT model currently handles gene-disease relationship prediction, the framework’s modular design allows for expansion to other entity-pairs like drug-gene, drug-disease, cell type-gene, and cell type-disease relationships. This expansion requires careful consideration of the computational approach – while fine-tuning for each relationship type is computationally intensive during training, it may prove more efficient during inference compared to alternatives like in-context learning or advanced prompting methods when processing the entire PubMed corpus of 39+ million abstracts. Additionally, expanding to new relationships will require developing comprehensive annotation schemas and guidelines to ensure consistent, high-quality training data. Additionally, our analysis has revealed that complex sentence structures with multiple clauses and nested relationships are currently underrepresented (~2.5%) in the training data we used. To address this, future improvements could incorporate targeted data augmentation strategies, including rule-based transformation of simpler sentences, back-translation for paraphrasing, and the use of external resources to generate diverse complex sentences. Secondly, the co-occurrence statistics score currently considers entities within the same sentence and does not account for publication quality, as we postulate that due to the large number of abstracts, statistical significance is an appropriate way to control for truth. However, especially for scientists applying our pipeline to a smaller text corpus, one could increase confidence in the quality of the included articles by thresholding or weighing based on journal impact factors derived from the journal name, which is readily available in the metadata. Another possibility would be to use citation counts as a measure of quality, but this data is not contained in the PubMed metadata and would require additional development. Regarding conference resolution, research from the BioNLP 2018 conference (Trieu et al., 2018) suggests that neural conference systems (e2e_coref Lee et al., 2017; NeuralCoref 4.0, 2021) can perform reasonably well on biomedical texts even without domain-specific features or in-domain embeddings. Finally, we used SciLinker to process PubMed abstracts, but the same strategy could be applied to full-text articles, increasing the number of co-occurrence sentences by orders of magnitude. Further refinements of SciLinker through expanded entity coverage, enhanced scoring methods, and broader text corpus analysis will improve SciLinker’s accuracy, reliability, and applicability in target discovery and credentialing.

## Conclusion

We have here presented SciLinker, a novel text mining approach that combines relationship extraction and co-occurrence-based statistical analysis to identify associations between genes, cell types, drugs, and diseases in biomedical literature. By analyzing co-occurrence patterns across large literature corpora and evaluating them with quantitative scores and statistical significance, SciLinker reliably extracts meaningful biomedical associations even from limited contextual information. While currently focused on PubMed abstracts, SciLinker’s modular design allows expansion to other entities and text sources, including full-text articles, clinical notes, and electronic medical records, making it a versatile tool for generating insights that can advance disease understanding and therapeutic development.

## Data Availability

Publicly available datasets were analyzed in this study. This data can be found: https://pubmed.ncbi.nlm.nih.gov/.

## Footnotes

^1^ https://ftp.ncbi.nlm.nih.gov/pubmed/baseline/

^2^ https://ftp.ncbi.nlm.nih.gov/pubmed/updatefiles/

^3^ https://www.citeline.com/
