# Supplementary material for: SciLinker: a large-scale text mining framework for mapping associations among biological entities
Source: Front Artif Intell. 2025 Mar 19;8:1528562. doi: 10.3389/frai.2025.1528562 (PMC11983328; doi:10.3389/frai.2025.1528562)
Supplement: Supplementary file 2 [file Presentation_1.pptx]

## Slide 1
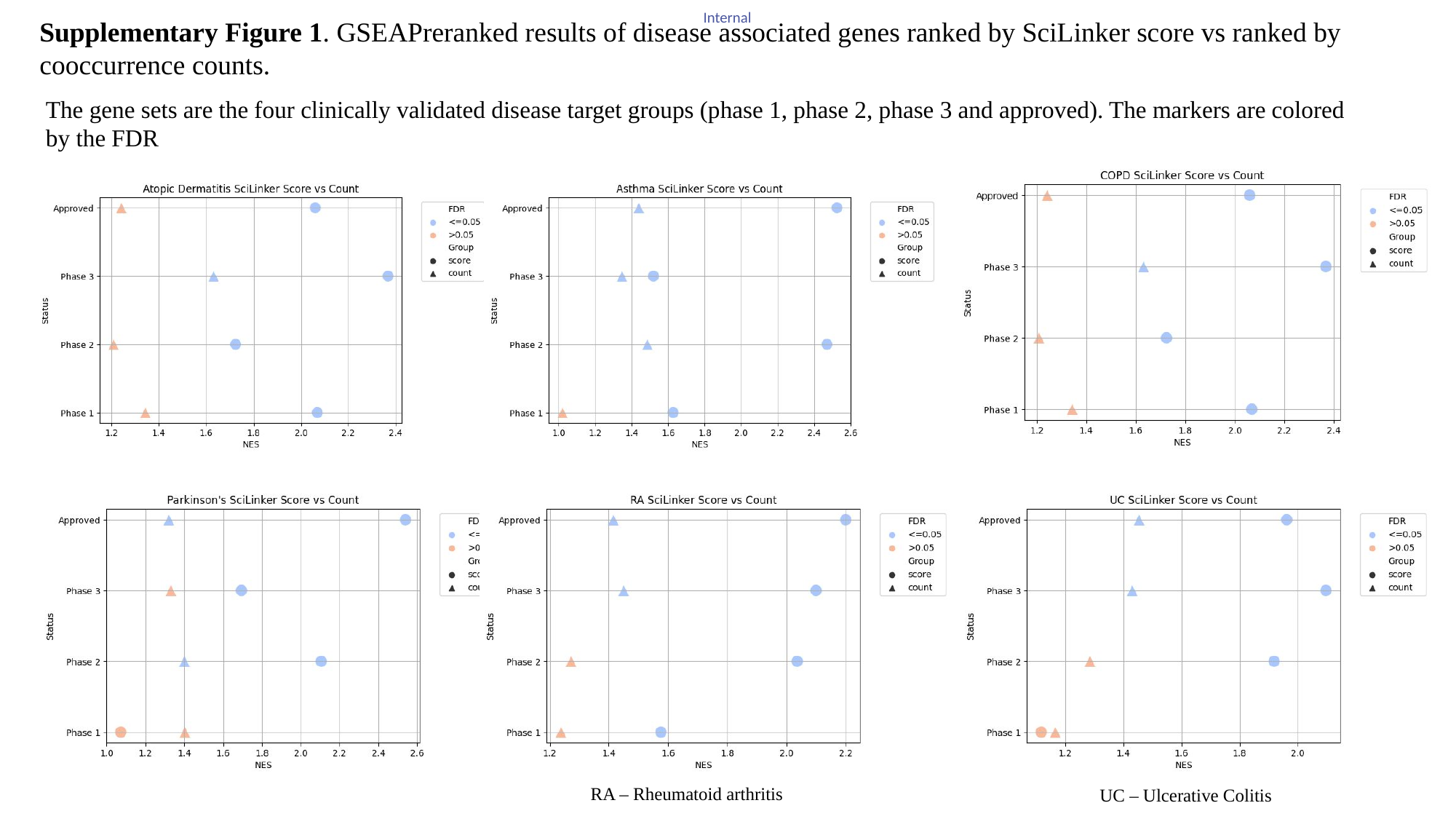

Supplementary Figure 1. GSEAPreranked results of disease associated genes ranked by SciLinker score vs ranked by cooccurrence counts.
The gene sets are the four clinically validated disease target groups (phase 1, phase 2, phase 3 and approved). The markers are colored by the FDR
RA – Rheumatoid arthritis
UC – Ulcerative Colitis
